# Recorded Loneliness and Adverse Outcomes in Older Acute Care Inpatients Receiving Psychiatric Assessment

**DOI:** 10.1002/gps.70052

**Published:** 2025-01-30

**Authors:** Moyenda Joseph, Kate Lockie, Agnes Mbazira, Robert Stewart

**Affiliations:** ^1^ South London and Maudsley NHS Foundation Trust London UK; ^2^ King's College London (Institute of Psychiatry, Psychology and Neuroscience) London UK

**Keywords:** hospitalisation, liaison psychiatry, loneliness, mortality

## Abstract

**Objectives:**

We investigated the prevalence of loneliness recorded during assessment of general hospital inpatients by older adult liaison psychiatry services and its associations with level of subsequent hospitalisation, emergency presentation and mortality.

**Methods:**

Data were drawn from a large south London mental healthcare provider of older adult liaison psychiatry services to four acute general hospitals. The sample comprised all patients receiving assessments from these services from 2007–2017. Recorded loneliness was ascertained from text fields via a bespoke natural language processing algorithm and, via a linkage with national hospitalisation data, was investigated as a risk factor for repeat emergency department (ED) attendance, inpatient days in the subsequent 12 months, and mortality.

**Results:**

In 11,631 patients assessed, loneliness was recorded in 11.2%. After adjustment for a range of demographic and health covariates, recorded loneliness was associated with an increased risk of ED attendance, but with lower mortality and, in survivors, with fewer hospitalisation days over a 12‐month follow‐up.

**Conclusions:**

Loneliness is recorded in over 10% of inpatients assessed by older adult liaison services and is likely to be present in substantially more. Lack of recording in more severe illness and/or cognitive disorders may explain associations with lower mortality and hospitalisation days. Its association with higher likelihood of repeat ED attendance suggests that loneliness should be considered more routinely in clinical assessments, possibly with formal screening.

**Summary**

- Loneliness is recorded frequently in general hospital inpatients receiving mental health assessments.
- Loneliness in these clinical settings is likely to be underestimated because it is not routinely screened for.
- The higher risk of emergency department re‐attendance in people recorded as being lonely suggests that it should be more often considered and recorded, and that interventions should be developed to provide and/or encourage more social support.

## 1 Introduction

Loneliness, often described as the subjective feeling of social isolation or lack of companionship, has emerged as a significant public health concern in recent years. A 2021–2022 survey found that 8.5% of people in England aged 75+ often or always felt lonely. However, some studies have suggested prevalences as high as 37% in older inpatients, compounded by visiting restrictions, limited staff contact, and relations with other inpatients disrupted by high turnover and inpatient mortality [1, 2, 3, 4]. With increasing concerns around social disconnection particularly in older adults, understanding the ramifications of loneliness on physical and mental health outcomes has become imperative. As well as associations with higher mortality, loneliness has been linked with worse cardiovascular risk and immune dysregulation as well as higher risk of dementia and depression [5, 6, 7, 8, 9]. Loneliness can increase the risk of misdiagnosed mental disorder, and lack of social support may impede progress and increase risk of relapse [10]. Relatively little is known about associations with hospitalisation and length of stay; however, increasing numbers of inpatients experiencing delayed discharge are likely to exacerbate all of the above complications [11].

As the global population ages, the need for comprehensive healthcare services that address the unique challenges faced by older adults has become increasingly critical. Patients aged over 65 occupy approximately two thirds of general hospital inpatient beds, and make up around 40% of all hospital admissions and approximately 15% of those patients who are discharged are readmitted within 28 days [12, 13] In addition to this, patients with concurrent psychiatric conditions are also at a higher risk of being readmitted and studies have shown that early involvement with liaison services results in fewer readmissions. Data collected by Mujic and colleagues highlighted the complexity of these patients and demonstrated that specialised older adult liaison services are essential in general hospitals with notable improvements in Health and Nation Outcome Scales (HoNOS) following liaison psychiatry involvement [14, 15].

Consequently, dedicated older adult liaison psychiatry input has become an increasingly prevalent service model provided to general hospital inpatients, recognising that the mental healthcare needs of older inpatients are better provided by specialist teams rather than by local community mental health or generic liaison services [16]. However, there has been relatively little large‐scale evaluation of information collected in this sector or evidence for outcome associations.

Taking advantage of a large mental healthcare data resource, incorporating granular information from older adult liaison services provided to four general hospitals over more than a decade, we investigated the prevalence of loneliness recorded during the psychiatric assessment in that context and its associations with level of subsequent hospitalisation, emergency presentation and mortality.

## 2 Materials and Methods

### 2.1 Data Source/Setting

Data were drawn from the South London and Maudsley NHS Foundation Trust (SLaM), a large and near‐monopoly provider of comprehensive mental healthcare to a geographic catchment area of four south London boroughs (Croydon, Lambeth, Lewisham, Southwark) and approximately 1.3 m residents. SLaM services have used fully electronic health records since 2006 and its Clinical Record Interactive Search (CRIS) platform was set up in 2008 to provide researcher access to full but de‐identified data from these records within a robust, patient‐led, security model [17, 18, 19]. CRIS mental healthcare data have been substantially enhanced since its development with external data linkages, including the national Hospital Episode Statistics (HES) data used for this study, and with a large number of natural language processing (NLP) algorithms to extract data from text fields, including recorded loneliness, the primary exposure for this study. CRIS at the Maudsley has received research ethics approval as a data resource for secondary analyses (Oxford Research Ethics Committee C, reference 23/SC/0257).

### 2.2 Sample and Measurements

Data were extracted for this study on all patients who received an assessment by one of the four older adult liaison services provided by SLaM between 1st January 2007 and 31st December 2017, taking the index date as the first recorded face‐to‐face contact during that period. The primary exposure, recorded loneliness, was ascertained from a specific NLP algorithm that has been described in previous publications and which, in summary, seeks to ascertain any description of the patient being lonely or reporting loneliness [20, 21, 22]. Data on hospitalisation outcomes were derived from HES which at the time of extraction contained CRIS‐linked files from all hospital admissions in England up to 31st March 2020. From these, dates of first emergency department (ED) attendance after the index date were calculated, as were the number of inpatient days in the 12 months after the index date. Date of death was additionally considered as an outcome.

The following covariates were also derived for this analysis, quantified at the index date: (i) age; (ii) gender; (iii) ethnic group (categorised into White, Black, Asian, Other, and Not Stated); (iv) neighbourhood deprivation (Index of Multiple Deprivation (IMD), a commonly used composite measure derived from the 2011 national census, applied to Lower Super Output Area, a standard administrative unit containing an average of 1500 residents, and categorised by quintiles); (v) psychiatric diagnosis (taken from structured fields in the record and the closest recorded instance to the index date; diagnoses are recorded by International Classification of Diseases 10th edition (ICD‐10) codes and were grouped according to the most frequent categories); (vi) level of recent mental health care receipt (number of active SLaM referral days in the 12 months prior to the index date); (vii) primary medical diagnosis from the index admission (extracted from linked HES files by ICD‐10 code and categorised into the most common systems represented) [23]. In addition, data were extracted on marital and co‐residency status (the latter ascertained by an NLP algorithm for “living alone”) for descriptive purposes; however, these were not used further in analyses due to strong associations with exposure status and the consequent risk of over‐adjustment.

### 2.3 Statistical Analyses

Having described the analysed sample, unadjusted analyses were carried out on exposure distributions by covariates and outcome categories. Cox proportional hazards models were used to investigate associations with time to ED attendance and mortality. Poisson regression models were used for hospitalisation days in the subsequent 12 months, restricting these analyses to the sub‐sample who had died during that period. Regression models included covariates in sequential stages: Model 1 unadjusted; Model 2 adjusting for age only; Model 3 adding gender, ethnic group, and IMD category; Model 4 adding psychiatric diagnosis and recent mental healthcare receipt; Model 5 adding primary diagnosis for the index hospitalisation. Results were presented as respective regression coefficients (hazard ratios, HRs, for Cox models; incidence rate ratios, IRRs, for Poisson models).

## 3 Results

The analysed sample comprised 11,631 patients receiving their first contact with older adult liaison psychiatry services. The sample had a mean (SD) age of 80.4 (8.0) at the index date and characteristics are summarised in Table 1. The most frequent age decade was 80–89 years, 56% were female, around three quarters were from a White ethnic group, and two thirds were separated or widowed. Dementia and delirium were the most common mental health diagnoses, and circulatory disorders were the most common specific category for primary medical diagnosis. By the end of the follow‐up period, 90% had died and 74% had at least one further ED presentation.

**TABLE 1:** Sample characteristics (*n* = 11,631).

Characteristic		% or mean (SD)
Age group (%)	60–69	12.1
	70–79	34.4
	80–89	41.3
	90+	12.2
Gender (%)	Female	56.2
	Male	43.8
Ethnic group (%)	White	76.4
	Black	11.4
	Asian	1.9
	Other	0.2
	Not known	10.1
Mean (SD) neighbourhood deprivation score		24.7 (10.4)
Marital status (%)	Separated/widowed	65.6
	Married/cohabiting	26.1
	Not known	8.3
Living alone (%)		41.7
Mental health diagnosis (%)	Dementia	24.1
	Delirium	25.4
	SMI	6.2
	Depression/anxiety	19.0
	Adjustment	9.7
	Other	6.3
	None	9.4
Hospital diagnosis (primary; %)	Neoplasm	6.2
	Psychiatry/Neurology	13.6
	Cardiovascular	14.7
	Respiratory	12.1
	Genito‐urinary	12.8
	External cause	11.3
	Other diagnosis	20.7
	Miscellaneous	8.6
Any mental healthcare contact in the preceding 12 m (%)		53.3
Mean (SD) days in hospital over following 12 m		42.6 (49.9)
ED presentation during follow‐up (%)		73.6
Mortality during follow‐up (%)		90.4

Recorded loneliness was present in 11.2% of the sample and its distributions by covariates and outcome groups are displayed in Table 2. In summary, loneliness was more common in older patients, in women compared to men, in separated/widowed patients or those living alone, in those from higher deprivation neighbourhoods, and in those with higher levels of prior mental healthcare contact. Significant heterogeneity of recorded loneliness was observed by ethnic group (with highest frequencies in White, Other, or unknown groups), by mental health diagnosis (with highest frequency in depressive/anxiety disorders), and by primary discharge diagnosis (with highest frequency in those with external or miscellaneous disorders). Considering outcomes, recorded loneliness was associated with future ED presentation, but was not associated with grouped hospitalisation days, and was more frequent in survivors than those who died during follow‐up.

**TABLE 2:** Distribution of recorded loneliness by sample characteristics and outcome groups.

Characteristic		Number	% loneliness	Chi‐square (df), *p*‐value
Age group	60–69	1407	10.4	
	70–79	4002	10.1	10.8 (1)
	80–89	4802	11.8	0.001
	90+	1420	13.2	
Gender	Female	6530	13.0	47.6 (1)
	Male	5097	8.9	< 0.001
Ethnic group	White	8881	11.7	
	Black	1330	7.7	20.3 (4)
	Asian	221	9.5	< 0.001
	Other	27	18.5	
	Not known	1172	11.4	
Neighbourhood deprivation (IMD, quintile group)	1 (least)	2304	8.6	
	2	2290	11.0	13.9 (1)
	3	2303	12.6	< 0.001
	4	2312	12.1	
	5 (most)	2275	11.9	
Marital status	Separated/widowed	7634	14.0	
	Married	3033	4.8	187 (2)
	Not known	964	9.2	< 0.001
Living alone	No	6782	6.5	359 (1)
	Yes	4849	17.7	< 0.001
Mental health diagnosis	Dementia	2801	9.0	
	Delirium	2951	7.8	
	SMI	716	11.7	184 (6)
	Depression/anxiety	2215	18.9	< 0.001
	Adjustment	1123	11.2	
	Other	737	12.1	
	None	1088	9.5	
Primary discharge diagnosis after hospitalisation	Neoplasm	719	7.9	
	Psychiatric/Neurological	1586	12.1	
	Circulatory	1711	8.8	
	Respiratory	1409	10.8	50.1 (7)
	Genitourinary	1486	10.6	< 0.001
	External	1313	14.6	
	Other diagnoses	2410	10.5	
	Miscellaneous	997	14.8	
SLaM activity last 12 m (days)	0	5437	9.9	
	1	1472	11.5	32.6 (1)
	2–15	1906	9.2	< 0.001
	16+	2816	14.9	
Hospitalisation days next 12 m	0–8	2442	11.9	
	9–20	2317	11.2	0.99 (1)
	21–36	2270	10.5	0.319
	37–64	2296	11.8	
	65–365	2306	10.6	
A&E presentation	No	3076	6.8	80.0 (1)
	Yes	8555	12.8	< 0.001
Death	No	1117	15.2	20.2 (1)
	Yes	10,514	10.8	< 0.001

Regression model output is displayed in Table 3. Recorded loneliness was associated with increased hazard of future ED presentation in all models, robust to adjustment; however, it was associated with lower rather than higher mortality hazard and, in survivors, with fewer hospitalisation days.

**TABLE 3:** Cox and Poisson regression analyses for associations of recorded loneliness with subsequent emergency department (ED) presentation, mortality, and 12‐month hospitalisation days.

	Hazard ratio (95% CI)		Incidence rate ratio (95% CI) 12‐month hospitalisation days^a^
	ED presentation	Mortality	
1. Unadjusted	1.19 (1.11–1.27)	0.81 (0.76–0.86)	0.888 (0.878–0.898)
2. Age	1.18 (1.11–1.26)	0.79 (0.74–0.84)	0.887 (0.877–0.897)
3. Model 2 plus gender, ethnicity, neighbourhood deprivation	1.18 (1.10–1.25)	0.79 (0.74–0.84)	0.895 (0.885–0.905)
4. Model 3 plus mental health diagnosis, prior mental health contact	1.17 (1.09–1.25)	0.80 (0.75–0.85)	0.911 (0.900–0.921)
5. Model 4 plus hospitalisation primary diagnosis	1.17 (1.10–1.25)	0.82 (0.77–0.87)	0.924 (0.913–0.934)

^a^ Restricted to the sub‐sample (*n* = 7304) surviving for at least 12 months.

## 4 Discussion

In a large cohort of older general hospital inpatients receiving liaison psychiatry assessment, we investigated the prevalence and correlates of recorded loneliness and associations with subsequent hospitalisation and mortality. Loneliness had been recorded in 11% of the sample and was associated with earlier repeat emergency department presentation; however, it was associated with lower mortality and in lower overall hospital bed days in those who survived at least 12 months after the original assessment.

As mentioned, research findings over a number of years have indicated worse health outcomes in people who are lonely and/or socially isolated. Physical health associations include increased risk of hypertension and metabolic syndrome potentially due to hypothalamic‐pituitary‐adrenal axis dysregulation or unhealthy lifestyle behaviours such as poor dietary choices, physical inactivity, smoking and excessive alcohol consumption [6, 24, 25, 26]. These unhealthy behaviours can exacerbate existing health conditions or lead to new health problems, increasing the likelihood of acute episodes that require ED attendance. In addition to cardiovascular effects, studies have also linked loneliness to compromised immune function and susceptibility to inflammatory disease, possibly mediated by reduced anti‐inflammatory, or increased pro‐inflammatory, pathway activities [27, 28]. Lower levels of social cohesion have been more specifically associated with lower antibody response to the COVID‐19 vaccination [7]. Despite this, loneliness is not routinely ascertained or recorded in general hospital care, hence the focus on a cohort receiving mental healthcare input because of this higher likelihood of consideration and the capacity to ascertain recorded loneliness from text fields in the source records. Significant heterogeneity was observed in recorded loneliness between broad primary hospitalisation diagnoses (Table 2) with highest rates in those with psychiatric/neurological, external, and miscellaneous diagnoses, and lowest rates in those with circulatory or neoplastic diagnoses. As well as mental health comorbidity, this may reflect reasons for hospitalisation which are associated with social isolation, such as falls, self‐neglect, and inadequate functional support. In addition, patients hospitalised with life‐threatening cancers or cardiovascular disorders may receive more support from friends and family as a consequence, or the severity of the condition may obscure any concerns about loneliness. However, these observations were only from unadjusted associations and a more focussed, in‐depth investigation would be required to tease out the likely complex patterns of multimorbidity in this cohort.

The increased risk of ED attendance associated with recorded loneliness in our results aligns with existing literature. One possibility is that loneliness‐induced physiological changes and/or exacerbation of physical health outcomes may contribute to a higher likelihood of acute health incidents requiring emergency care. Chamberlain et al, 2022 also emphasized that lonely individuals are more likely to utilise emergency services, even after controlling for various health conditions, suggesting that the association is independent of those health conditions and suggesting an alternative correlation [29]. However, studies have also shown that loneliness often coexists with mental health conditions such as depression and anxiety which can exacerbate physical health outcomes [30]. A study by Hawkley and Cacioppo (2010) reported that loneliness is a significant predictor of depressive symptoms, which can, in turn, lead to neglect of self‐care and management of chronic diseases subsequently leading to more hospital admissions [31]. It is also noteworthy in our own study that loneliness varied more strongly between mental health than physical health diagnoses. While heightened health anxiety may prompt individuals to seek medical attention for more minor ailments, socially isolated individuals may also have fewer social supports to assist with early symptom recognition and management, leading to delays in seeking medical help until conditions become severe and requiring emergency care [32]. Finally, the association of recorded loneliness with lower mortality (discussed further below) might result in increased ED attendance due to preferential survival.

As well as long‐recognised associations with depression, loneliness is also recognised to be a significant predictor of cognitive decline and dementia in later life. For example, Wilson et al. (2007) found that the risk of Alzheimer's disease more than doubled in individuals who reported feeling lonely compared to those who did not. Furthermore, Russell et al. (1997) found that loneliness was associated with both an increased likelihood of, and reduction in time to nursing home admission over a 4‐year period in a cohort of around 3000 older rural US residents [8, 33]. Consistent with these adverse health associations, reduced social networks and loneliness as a component of these, have been repeatedly found to be significant predictors of mortality. Contrary to expectations, however, our study found lower rather than higher mortality associated with loneliness. A potential explanation is that the observed higher likelihood of ED attendance resulted in higher likelihood of disorder detection and intervention, consequently contributing to reduced mortality rates. However, it is also likely that the most severely ill individuals—in particular, those referred for psychiatric input because of delirium states—were less likely to report loneliness and/or less likely to have loneliness recorded in their assessments, therefore skewing the exposure towards those at lower risk of mortality. Furthermore, although loneliness may be a risk factor for dementia, it may be less likely to be recorded as a consideration once dementia has become manifest and/or is more severe and/or is accompanied by delirium or behavioural symptoms, accounting for the association with shorter rather than longer total hospitalisation. Finally, lonely individuals might also underreport symptoms due to lack of social contact, or fear burdening others, accounting for shorter hospital stays.

Strengths of this study included the large sample, the naturalistic data, and the novel measure of recorded loneliness which has been found to be an important outcome predictor in other cohorts [20, 21, 22]. It is important to bear in mind the nature of the sample when drawing conclusions, as this comprised older inpatients from general hospitals who had received liaison psychiatry input. These should clearly not be viewed as representative of older inpatients more widely; however, with increasingly standard provision of liaison services of this nature, the clinical cohort should be reasonably representative of referrals at other sites. The primary exposure, it should be emphasised, is loneliness that has been recorded, which clearly is likely to be an underestimate, since many instances may not be enquired about. However, the prevalence is reasonably high, and the derived variable therefore defines a risk group which warrants both further attention and perhaps more assertive screening for cases of loneliness which would otherwise have been missed. Hospitalisation incidence and duration and mortality are three outcomes amongst many that could have been chosen, and future research may wish to investigate this more widely, as well as seek to include a broader range of covariates for adjustment. The cohort was assembled from healthcare data before the COVID‐19 pandemic in order to avoid complications in interpretation arising from the substantial disruptions to service provision around that time. Further replication is therefore warranted in post‐pandemic cohorts to clarify any more recent changes in loneliness prevalence and impact.

## 5 Conclusions

While extensive research has examined the association between loneliness and various physical and mental health outcomes, little attention has been directed towards its potential impact on hospitalisation or its occurrence in older inpatients with mental health conditions who may be particularly vulnerable. Understanding this relationship is crucial as recurrent hospitalisation, even if not prolonged, not only imposes significant financial burden of healthcare systems but may also exacerbate any associated psychological distress and hamper longer‐term recovery. By understanding how loneliness may affect these critical aspects of healthcare delivery, we can develop targeted interventions to mitigate its impact and improve patient outcomes. At the very least, it is something worth enquiring about more commonly in routine clinical practice.

## Conflicts of Interest

RS declares research support received within the last 3 years from GSK. No other interests to declare.

## Data Availability

The data that support the findings of this study are available on request from the corresponding author. The data are not publicly available due to privacy or ethical restrictions.
